# Supplementary material for: Longitudinal co‐development of mental and cardio‐metabolic health from childhood to young adulthood
Source: J Child Psychol Psychiatry. 2025 Oct 17;67(3):413–24. doi: 10.1111/jcpp.70065 (PMC12883583; doi:10.1111/jcpp.70065)
Supplement: Supplementary file 1 — Appendix S1. Cardio‐metabolic risk markers – measurement details. Appendix S2. The intuition behind the (RI)‐CLPM. Appendix S3. The ALT‐SR model. Figure S1. Cardio‐metabolic risk markers – distributions. Figure S2. CLPM and RI‐CLPM. Figure S3. Cross‐lag associations between maternal reports of depressive symptoms and cardio‐metabolic risk factors. Figure S4. ALT‐SR (vs. RI‐CLPM) results. [file JCPP-67-413-s001.docx]

**Supplementary materials**

**Longitudinal co-development of mental and cardio-metabolic health from childhood to young adulthood**

Table of contents

[Appendix S1 | Cardio-metabolic risk markers – measurement details 2](#_Toc199152193)

[Figure S1 | Cardio-metabolic risk markers – distributions 3](#_Toc199152194)

[Appendix S2 | The intuition behind the (RI)-CLPM 4](#_Toc199152195)

[Figure S2 | CLPM and RI-CLPM 5](#_Toc199152196)

[Figure S3 | Cross-lag associations between maternal reports of depressive symptoms and cardio-metabolic risk factors 6](#_Toc199152197)

[Appendix S3 | The ALT-SR model 7](#_Toc199152198)

[Figure S4 | ALT-SR (vs. RI-CLPM) results 8](#_Toc199152199)

## Appendix S1 | Cardio-metabolic risk markers – measurement details

Besides, fat mass index (FMI), ten alternative cardio-metabolic risk markers were examined in secondary analyses. These included: lean mass index (LMI), body mass index (BMI), waist circumference, android fat mass, high-density lipoprotein (HDL) and low-density lipoprotein (LDL) cholesterol levels, triglycerides, insulin, and C-reactive protein (CRP). These were measured as follows:

Total body fat and lean mass (g) were derived from whole body dual energy X-ray absorptiometry (DXA) scans at six time points (at median ages of 9.8, 11.8, 13.8, 15.4, 17.8, and 24.5 years).^1^ Android fat mass (g) was extracted from the last four DXA scans. Child weight (kg) and height (cm) were either measured during the same six research visits or reported in questionnaires (at median ages of 10.7, 12.8, 16, 17 years)^2^. Participants total body fat mass, lean mass and weight were then divided by squared height to obtain FMI, LMI and BMI respectively (kg/m^2^). Waist circumference (cm) was measured six times (at median age of 9.8, 10.6, 11.8, 12.8, 15.4, and 24.5 years). HDL and LDL cholesterol, triglycerides, insulin and CRP were measured in non-fasting (at 9.8 years) or fasting (at 15.4, 17.8 and 24.5 years) blood samples.

##
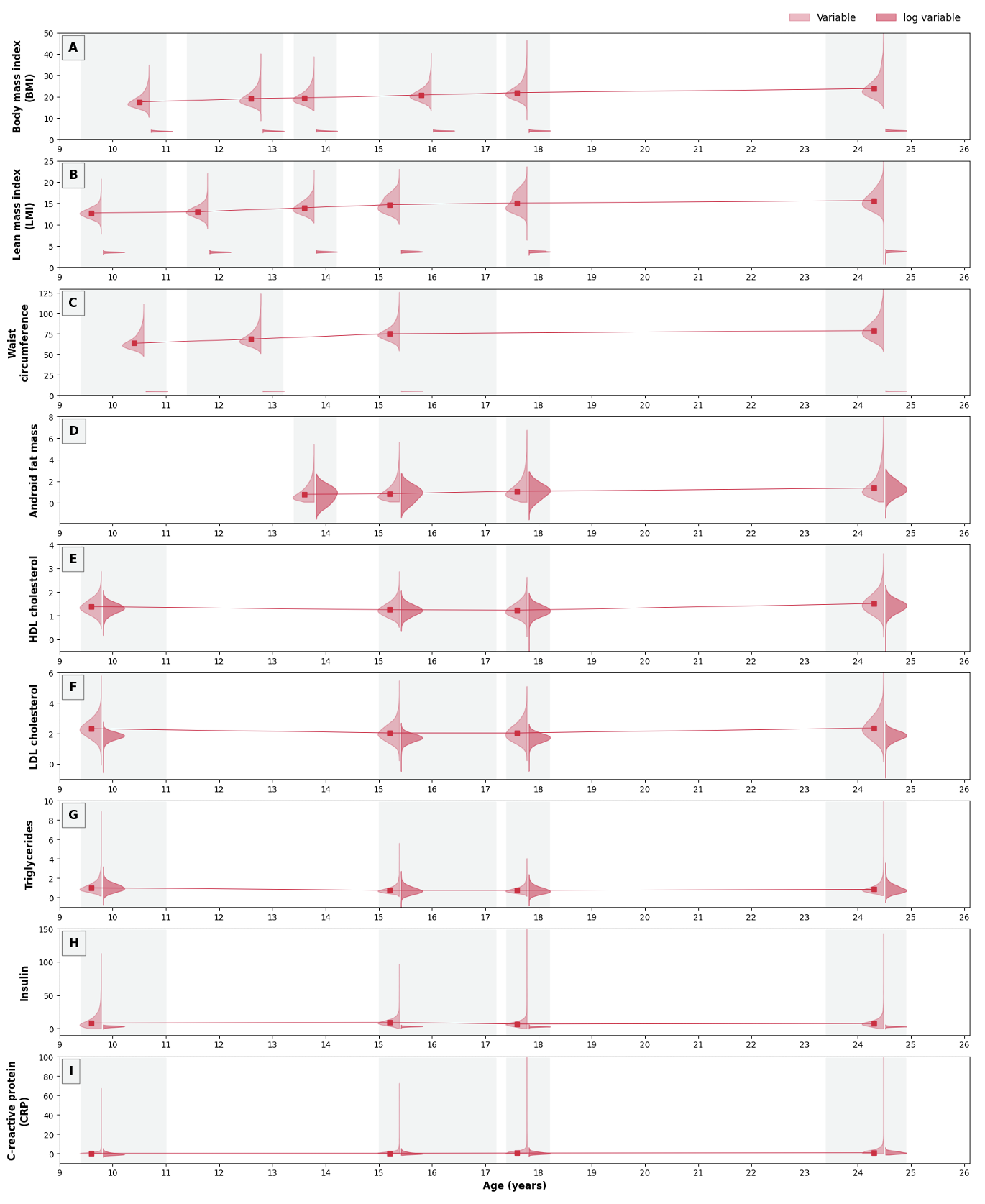
Figure S1 | Cardio-metabolic risk markers – distributions

The distribution of observed values for all alternative cardio-metabolic risk markers used in secondary analyses is presented on the y-axis against measurement time (x-axis). These include (A) body mass index, (B) lean mass index, (C) waist circumference, (D) android fat mass, (E) high-density lipoprotein cholesterol, (F) low-density lipoprotein cholesterol, (G) triglycerides, (H) insulin, and (I) C-reactive protein. In the violin plots, lighter colors are used to represent the original value distributions, while darker colors represent the same variable distributions and after data transformation was applied (i.e., log transformation). The line graph connects the median points (in the original data scale) at each timepoint.

## Appendix S2 | The intuition behind the (RI)-CLPM

If we conceptualize human health as a “dynamic system”, which evolves over time by repeatedly driving a state (at time *t*) to another state (at time *t* + 1), we can then understand each “health state” as a function of *a)* their prior values, *b)* external inputs to the system and *c)* the transition rules or “updating mechanisms” that govern change.

An example of transition rule is *persistence* (also known as inertia or self-similarity). This is the tendency for a construct to retain its state over time, until something (i.e., some external input) changes it. In traditional cross-lag panel modeling (CLPM)^3^, persistence is quantified by autoregressive (AR) terms which are simply regression coefficient describing the relationship between a state and its previous values (e.g., depression at time 2 ~ depression at time 1).

Another set of transition rules, perhaps the most interesting ones from a scientific standpoint, are *reciprocal relationships*. Reciprocal relationships describe patterns of feedback across multiple variables, or the “tendency for constructs to form causal loops in which one construct initiates subsequent events that further change the original construct at a later period”. For example, the onset of a depressive episode may trigger a change in body weight (e.g., because of altered inflammatory processes or changes in lifestyle) which in turn influences future states of depression (e.g., by driving self-esteem, mood or social isolation). Reciprocal relationships are also quantified in CLPMs by cross-lag (CL) regression coefficients (e.g., weight at time 2 ~ depression at time 1; *Figure S2-A*).

***The random-intercept CLPM (RI-CLPM): controlling for unobserved heterogeneity***

The traditional CLPM we described so far (i.e., including AR and CL terms), enjoyed long-standing popularity in psychological and epidemiological research, thanks its intuitive ability to embody core notions of system dynamics (i.e., persistence and reciprocal influence). However, this simple model has been criticised by many on account of one major methodological shortcoming: the inability to disentangle between-person effects from within-person effects, or, in other words, the lack of control for *unobserved heterogeneity*.

The random-intercept CLPM (RI-CLPM)^4^ is among the more recent extensions of this framework, specifically developed to address this issue.

The RI-CLPM supposes a latent factor for each variable series (e.g., η*_DEP_* and η*_CMR_*; *Figure S2-B*), i.e. the “random intercepts”. These are meant to represent the collection of unmeasured confounding factors which are stable over time within people but vary from one person to the other. We refer to this concept as “unobserved heterogeneity” (but it is also known as “time-invariant confounding”, “stable trait factor”, or “unit fixed effects”). For example, sex, socio-economic status or genetic predisposition may be responsible for persistently higher adiposity levels across development in certain people. These stable between-person differences should not be confounded with the within-person dynamics that we mean to quantify in AR and CL terms. For this reason, the inclusion of random intercepts is a major strength of the RI-CLPM, over to the traditional CLPM, as it allows us to infer the within-person relationships between variables, that would be otherwise confounded by stable interindividual differences.

The factor loadings of these time-invariant latent factors are typically set to 1 (similarly to random intercepts in fixed-effects models).

***The generalized CLPM (gCLPM): relaxing stationarity assumptions***

Another recent alternative to traditional CLPM, is the generalized CLPM (gCLPM) framework^5^. The gCLPM introduces two further extensions to the RI-CLPM, which provide a way to relax the assumption of stationarity, i.e., that both between- and within person effects are constant over time. In summary the model:

1. Deals with unobserved *time-varying* confounders, by allowing freely estimated *η* factor loadings (“time-varying unit effects” as also mentioned in the previous paragraph). This added flexibility is important, for example, to account for major life transition periods, such as puberty, which may introduce interindividual differences (i.e., unobserved heterogeneity) that are not constant but rather have different effects across time (e.g., at age 10 vs. age 15).
2. Expands the range of temporal dynamics that the model can capture, by introducing *moving average* (MA) autoregressive and cross-lagged terms. These are time-varying coefficients, which act as direct effects of the residuals of each observed variable (known as “random impulses”). The implication of estimating these additional terms is that the influence of a predictor can be decomposed into a) a component that is stable over time, and b) a component that can differ over the various time waves. For example, adiposity may have a small influence on future depression in early adolescence but became a stronger contributor as people age.

Note that the gCLPM is not free of criticisms (see for example Usami, 2021^6^), and it is important to note how the increased flexibility may come at the cost less robust modelling results that are considerably more complex to interpret. Nonetheless, for readers who are interested in more complex characterizations of the range of dynamic processes underlying these relationships, we provide the gCLPM estimates for all variable of interests on the project web-application.

##
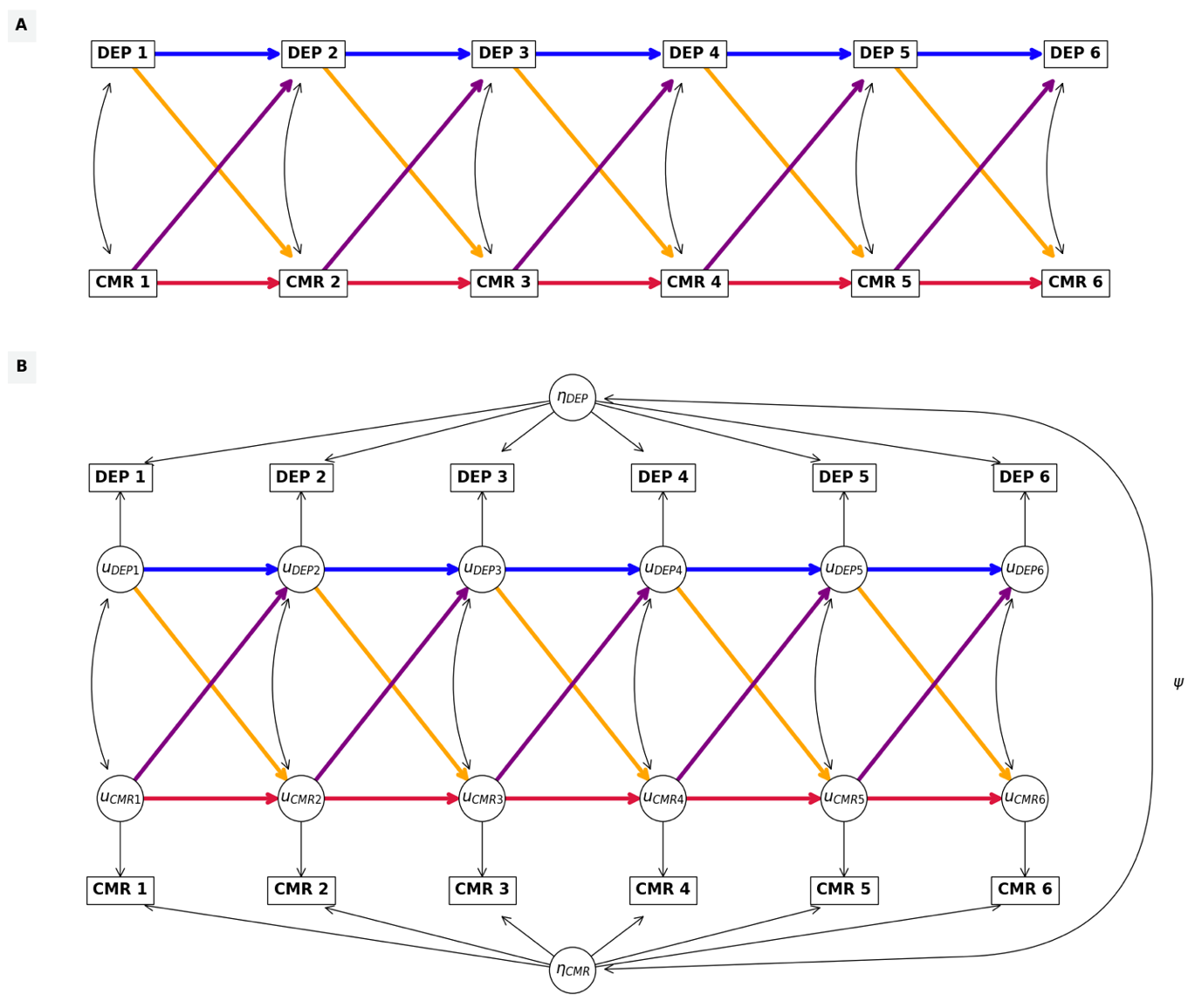
Figure S2 | CLPM and RI-CLPM

Graphic representation of (A) the traditional CLPM and (B) the RI-CLPM, described in *eMethods2*. Autoregressive (AR) terms are represented in blue and red, for depression (DEP) and cardio-metabolic risk (CMR) respectively. Cross-lag (CL) terms are depicted in yellow and purple, for depression to CMR and CMR to depression respectively.

##
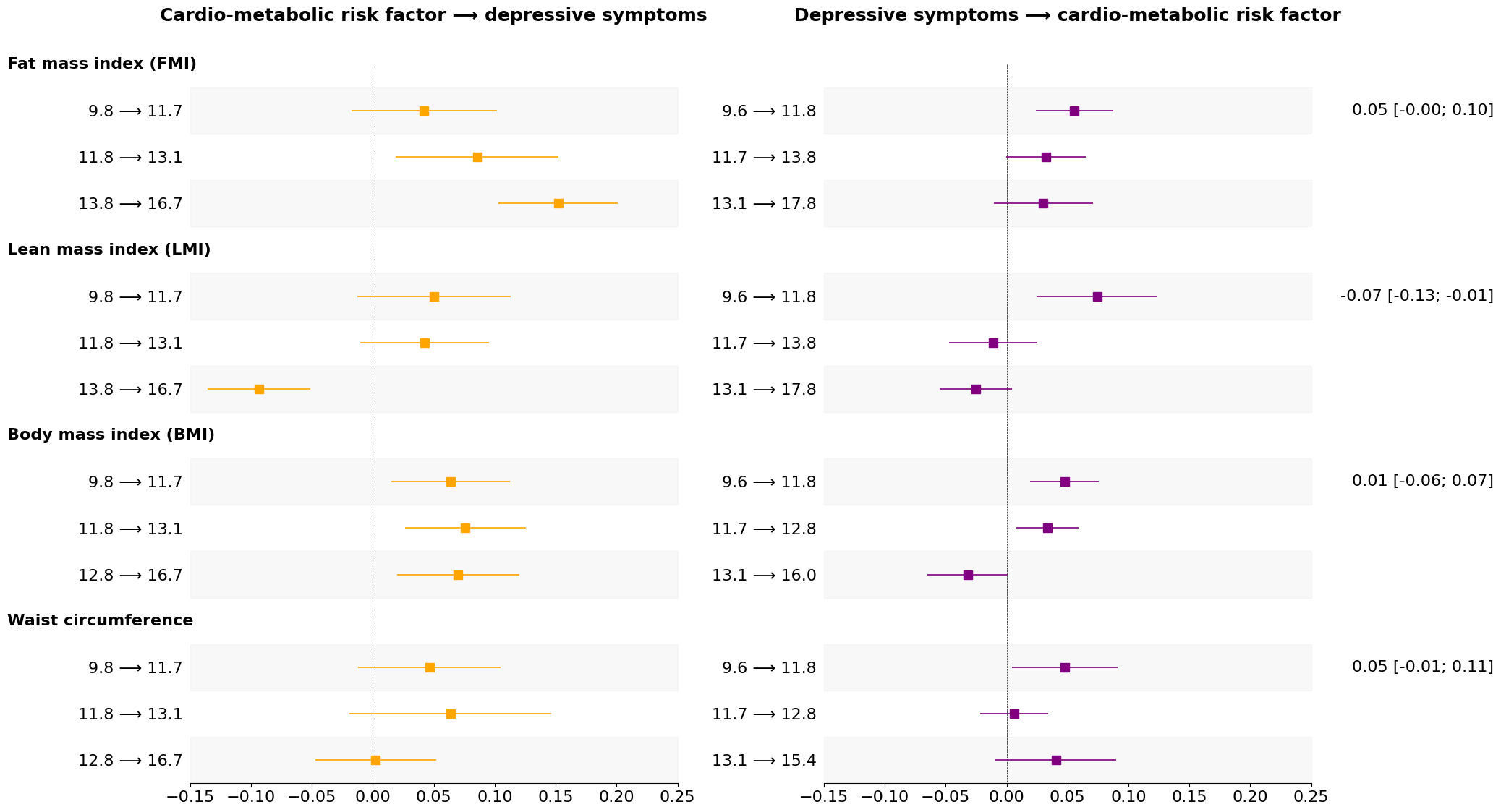
Figure S3 | Cross-lag associations between maternal reports of depressive symptoms and cardio-metabolic risk factors

The standardised regression coefficients for the within-person cross-lag associations (and their 95% confidence intervals) are presented along the x-axis, for each cardio-metabolic risk factor examined, listed on the y-axis. The temporal lag each estimate refers to is specified in years on the y-axis. Association estimates from cardio-metabolic risk factor to lagged depressive symptoms are presented in yellow on the left and those between depressive symptoms and lagged cardio-metabolic risk factor are shown in purple on the right. In the last column on the right of the graphs, the estimated correlation coefficient between the random intercepts of each construct (and its 95% confidence interval) is reported.

## Appendix S3 | The ALT-SR model

**Model description and rationale**

The Autoregressive Latent Trajectory Model with Structured Residuals (ALT-SR) is alterative modeling approach that extends the traditional CLPM framework. Like the RI-CLPM (see Appendix S2 for details) the ALT-SR allows for the separate estimation of within- and between-person effects over time. In other words, the goal in an ALT-SR model (like the RI-CLPM) is to disentangle stable, trait-like differences between individuals from dynamic, state-like changes within individuals across repeated measurements.

Compared to the RI-CLPM, ALT-SR explicitly models developmental trajectories (i.e. latent random *slopes*) in addition to the stable trait-like between-person differences (i.e., the latent random *intercepts* that give the RI-CLPM its name). This allows to capture more complex longitudinal patterns in the data.

For our exploratory follow-up analyses, we fit an ALT-SR model with linear latent growth (i.e., a latent factor for each variable series, γ*_DEP_* and γ*_CMR_*, with factor loading set to [0, 1, … *n_obs_* –1] ). The model was fit using lavaan version 0.6-19, and the [scripts](https://github.com/SereDef/comorb-longit-project/blob/main/1.2.ALTSR.R) are publicly available on the project GitHub repository.

However, the added complexity of ALT-SR comes at the cost of more parameters that need to be estimated, which may lead to known identification/convergence issues^7^. This was unfortunately the case for most analyses in our data.

**ALT-SR Results**

In a few cases, the ALT-SR model failed to converge entirely (e.g., for BMI, HDL and LDL cholesterol, as well as most of the models using mother-reports of depression). When the ALT-SR model did converge, improper solutions (i.e., Heywood cases, such as negative variances or >1 correlations) were much more common compared to the RI-CLPM (e.g., for android fat mass, and most lipid measures). These convergence issues especially affected analyses that relied on fewer (i.e. 4) time points, which is expected since the ALT-SR does require more time points for stable estimation (compared to the RI-CLPM). It is worth noting however that, when the ALT-SR did converge, it typically showed better fit performance than the RI-CLPM.

When we exclude models that either did not converge or that showed symptoms of unhealthy convergence for a subset of parameters, only the relationship between self-reported depression and FMI / LMI could be described by the ALT-SR. These results are reported below and represented in Figure S4 (in comparison with the corresponding RI-CLPM models).

The cross-lag path estimates describing the relationship between self-reported depression and FMI / LMI were relatively similar across ALT-SR and the RI-CLPM model, especially for the depression to fat / lean mass estimates (FMI average β [range] = 0.03 [0.02; 0.04], SE = 0.01; LMI average β [range] = –0.05 [–0.08; 0.01], SE = 0.01). Interestingly however, a slightly different pattern emerged from the ALT-SR compared to the RI-CLPM for the fat / lean mass to depression CL effects. These estimates were consistent in their direction (FMI average β [range] = 0.09 [0.01; 0.18], SE = 0.04; LMI average β [range] = –0.13 [–0.30; 0.03], SE = 0.03) but, while in the RI-CLPM they would “peak” between 13 to 15 years and then remain stable for the rest of follow-up, in the ALT-SR model they rather show a (linear) increase over time for the entire duration of follow-up (with peak estimate observed between 18 and 24 years).

Interestingly, in the ALT-SR the correlations among each construct’s random intercept and slope (indicating the portion of their relationship that is best explained by between-person differences in trait-like and in trajectories of depression and FMI/LMI) were following the directions of the CL effects (see Figure S4 for these estimates and their 95% CI).

As mentioned above, these models showed excellent model fit:

Depression – FMI: χ^2^(30) = 175.74, *p*<0.001; RMSEA [95%CI] = 0.025 [0.021-0.028]; CFI = 0.996; TLI = 0.991; SRMR = 0.012

Depression – LMI: χ^2^ (30) = 432.35, *p*<0.001; RMSEA [95%CI] = 0.041 [0.038-0.044]; CFI = 0.986; TLI = 0.969; SRMR = 0.030

**Generalisability considerations**

These results are very insightful and surely warrant further examination. Ideally, this examination should be powered by longitudinal datasets which are comparably large but offer a higher number of (more frequent) measurement occasions, to ensure successful parameter estimation. However, an important consideration to note is that more complex models such as the ALT-SR, come with a higher tendency to overfit the data. Despite the large sample size, the analyses presented above are based on a single cohort and need to be replicated before they can be effectively translated into clinical practice. Moreover, because of the long temporal span of this study, unbalanced loss to follow-up may have reduced data variability, further worsening the risk of overfitting (despite our best efforts to account for selection bias in parameter estimation).

##
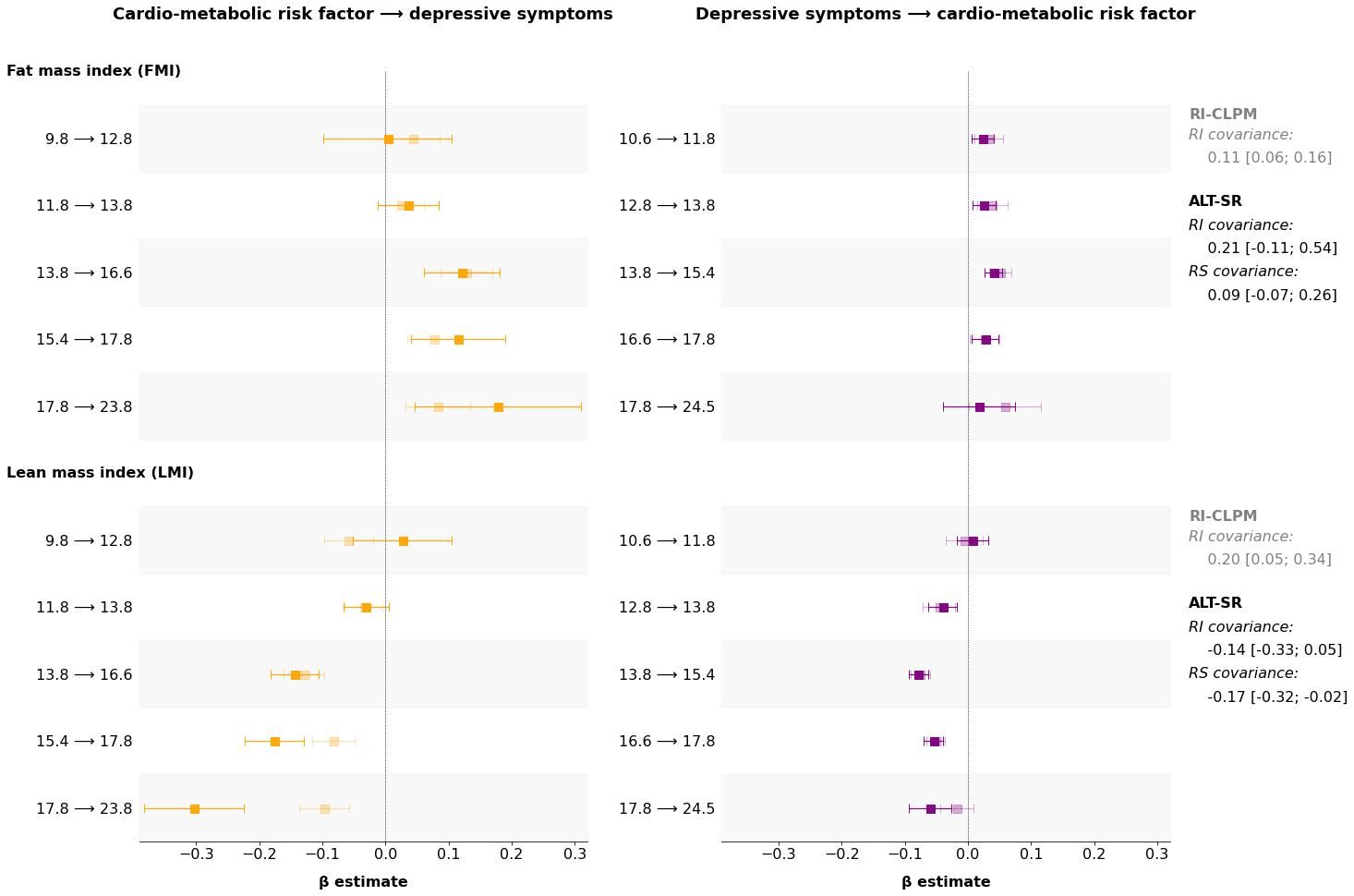
Figure S4 | ALT-SR (vs. RI-CLPM) results

The standardised regression coefficients for the within-person cross-lag associations (and their 95% confidence intervals) are presented along the x-axis. The two cardio-metabolic risk factors for which the ALT-SR showed healthy convergence (i.e. FMI and LMI) are listed on the y-axis. The matching terms estimated in the RI-CLPM are also displayed with higher transparency (there correspond to those depicted in Figure 3).

Similar to Figure 3, the temporal lag each estimate refers to is specified in years on the y-axis. Association estimates from cardio-metabolic risk factor to lagged depressive symptoms are presented in yellow on the left and those between depressive symptoms and lagged cardio-metabolic risk factor are shown in purple on the right.

The last column on the right of the graphs reports the estimated correlation coefficient between the random intercepts of the RI-CLPM (with its 95% confidence interval) in grey and that between the random intercepts and the random slopes of the ALT-SR (with their 95% confidence intervals) in black.

**References**

1. Dangardt F, Charakida M, Georgiopoulos G, et al. Association between fat mass through adolescence and arterial stiffness: a population-based study from The Avon Longitudinal Study of Parents and Children. *The Lancet Child & Adolescent Health.* 2019;3(7):474-481.

2. O'Keeffe LM, Kearney PM, Greene RA, et al. Maternal Alcohol Use during Pregnancy and Offspring Trajectories of Height and Weight: A Prospective Cohort Study. 2014.

3. Newsom JT. *Longitudinal structural equation modeling: A comprehensive introduction.* Routledge; 2015.

4. Hamaker EL, Kuiper RM, Grasman RP. A critique of the cross-lagged panel model. *Psychol Methods.* 2015;20(1):102-116.

5. Zyphur MJ, Allison PD, Tay L, et al. From Data to Causes I: Building A General Cross-Lagged Panel Model (GCLM). *Organizational Research Methods.* 2020;23(4):651-687.

6. Usami S. On the Differences between General Cross-Lagged Panel Model and Random-Intercept Cross-Lagged Panel Model: Interpretation of Cross-Lagged Parameters and Model Choice. *Structural Equation Modeling: A Multidisciplinary Journal.* 2021;28(3):331-344.

7. Orth U, Clark DA, Donnellan MB, Robins RW. Testing prospective effects in longitudinal research: Comparing seven competing cross-lagged models. *Journal of personality and social psychology.* 2021;120(4):1013.
